# Enhanced Product Recovery from Glycerol Fermentation into 3-Carbon Compounds in a Bioelectrochemical System Combined with *In Situ* Extraction

**DOI:** 10.3389/fbioe.2016.00073

**Published:** 2016-09-26

**Authors:** Hugo Roume, Jan B. A. Arends, Camar P. Ameril, Sunil A. Patil, Korneel Rabaey

**Affiliations:** ^1^Faculty of Bioscience Engineering, Center for Microbial Ecology and Technology (CMET), Ghent University, Gent, Belgium

**Keywords:** bioelectrochemical systems, resource recovery, *in situ* extraction, 1,3-propanediol, propionate

## Abstract

Given the large amount of crude glycerol formed as a by-product in the biodiesel industries and the concomitant decrease in its overall market price, there is a need to add extra value to this biorefinery side stream. Upgrading can be achieved by new biotechnologies dealing with recovery and conversion of glycerol present in wastewaters into value-added products, aiming at a zero-waste policy and developing an economically viable process. In microbial bioelectrochemical systems (BESs), the mixed microbial community growing on the cathode can convert glycerol reductively to 1,3-propanediol (1,3-PDO). However, the product yield is rather limited in BESs compared with classic fermentation processes, and the synthesis of side-products, resulting from oxidation of glycerol, such as organic acids, represents a major burden for recovery of 1,3-PDO. Here, we show that the use of an enriched mixed-microbial community of glycerol degraders and *in situ* extraction of organic acids positively impacts 1,3-PDO yield and allows additional recovery of propionate from glycerol. We report the highest production yield achieved (0.72 mol_1,3-PDO_ mol^−1^_glycerol_) in electricity-driven 1,3-PDO biosynthesis from raw glycerol, which is very close to the 1,3-PDO yield reported thus far for a mixed-microbial culture-based glycerol fermentation process. We also present a combined approach for 1,3-PDO production and propionate extraction in a single three chamber reactor system, which leads to recovery of additional 3-carbon compounds in BESs. This opens up further opportunities for an economical upgrading of biodiesel refinery side or waste streams.

## Introduction

Biodiesel production, as a promising alternative and renewable fuel, has continuously grown over the past decade (Clomburg and Gonzalez, 2013). However, growth in its production has been less than the anticipated target and has increased at a slower pace during the last few years (Yang et al., 2012). According to an internal review published by BP Company, world biofuels increased by 0.9% in 2015, the slowest rate of growth since output declined in 2000 (BP Statistical Review of World Energy, 2016). The main reason is its relatively high production cost. For every tone of biodiesel produced by transesterification of animal or vegetable oils, 100 kg of crude glycerol is coproduced (Mattam et al., 2013). The expansion of the biodiesel industry has, thus, created a surplus of glycerol, resulting in an abundance of waste glycerol now considered as a waste stream with associated disposal costs and a concomitant decrease in its overall market price. For instance, during 2004–2011, the biodiesel production has increased by 25 times and the market price of crude glycerol (80% pure) has decreased by a factor three in the USA (Clomburg and Gonzalez, 2013). Therefore, it is of major importance to find a recycling solution for this waste glycerol to make biodiesel production more sustainable and economically viable.

There are several approaches for direct utilization of crude glycerol or to convert it into higher-value products by means of biotechnological or chemical processes (Li et al., 2013). Compared with direct application and chemical transformation, microbial conversion of glycerol into other 3-carbon compounds by oxidation or reduction processes is a viable alternative that avoids certain disadvantages, such as low product specificity, high-energy input, and extensive pretreatment requirements (Li et al., 2013). Particularly, the most promising approach is to use glycerol as a substrate for bioconversion into 1,3-propandiol (1,3-PDO) *via* the intermediate 3-hydroxypropionaldehyde (3-HPA).

A wide variety of pure and engineered microorganisms have been used for glycerol conversion into value-added products (Li et al., 2013). For example, an engineered strain of *Escherichia coli* has been able to reach the highest product yield (1.09 mol_1,3-PDO_ mol^−1^_glycerol_), productivity (2.61 g L^−1^h^−1^), and high final 1,3-PDO concentration (104.4 g L^−1^) from glycerol (Tang et al., 2009). Compared with pure cultures, the use of a mixed-microbial community considerably decreases the production cost of a bulk chemical by working with simplified medium and under non-sterile conditions. Mostly, mixed cultures from different environmental sources such as sediments, sewage sludge, compost, decaying straw, mud, anaerobic distillery wastewater, and soil samples have been used directly for 1,3-PDO production (Dietz and Zeng, 2014). To the best of our knowledge, neither pre-selection nor enrichment of a specialized microbial community converting glycerol into 1,3-PDO has been reported in the literature.

The recovery of glycerol into 1,3-PDO has mainly been reported *via* anaerobic fermentation using a mixed-microbial culture (Dietz and Zeng, 2014; Moscoviz et al., 2016). One of the emerging approaches for resource recovery from wastes is the use of bioelectrochemical systems (BESs) that exploit microorganisms to catalyze redox reactions in an electrochemical cell (Arends and Verstraete, 2012). With BESs, electrical current can be used to drive fermentation toward the production of valuable chemicals from industrial wastes or side-streams, such as glycerol (Dennis et al., 2013). Imposing a cathodic current on a mixed-microbial consortium fermenting glycerol has been shown to stimulate the conversion of glycerol into 1,3-PDO (Zhou et al., 2013). Cathodic current is considered to favor microbial reduction processes by enhancing cells’ ability to regenerate NAD^+^ into NADH and is, thus, able to alter fermentation profiles (Dennis et al., 2013). Indeed, a critical NAD^+^/NADH ratio higher than 4, has previously been correlated with a high 1,3-PDO productivity and high specific growth rate of *Klebsiella pneumoniae* (Du et al., 2006). However, as evident in many fermentation processes using a mixed-microbial community, side-products are often generated (Marshall et al., 2012). During glycerol fermentation, pyruvate, the end product of the glycolysis pathway will compete with 3-HPA for NADH-oxidoreductase to form various organic acids as by-products, such as propionic acid (Dennis et al., 2013). A concomitant decrease in the yield of 1,3-PDO has indeed been found with an increase in propionic acid production in a BES fed with glycerol (Zhou et al., 2013).

In order to enhance the overall product recovery from a glycerol fermentation process into 1,3-PDO, by-products formed in the pyruvate reduction pathway should, thus, be maintained at the lowest level, stopped from being formed, or removed from the fermentation broth. Andersen et al. (2014) have recently reported an approach to *in situ* extract carboxylates generated during bioelectrochemical fermentation *via* membrane electrolysis across an anion exchange membrane (AEM) in BESs. Here, we hypothesize that the use of this BES-based carboxylate extraction technology in combination with the use of an enriched glycerol degrading mixed-microbial community as an inoculum should allow us to reach an optimized 1,3-PDO production yield and enhanced recovery of valuable by-products, such as carboxylates, generated during the electro-fermentation of glycerol.

## Materials and Methods

### Enrichment of a Specialized Glycerol-Degrading Microbial Community

A floating sludge sample from the first basin of the wastewater treatment plant of an oleochemical industry (Oleon NV, Ertvelde, Belgium) was used to enrich the specialized glycerol-degrading microbial community. The selection of this community was motivated by the high load of chemical oxygen demand (COD) of 3716 kg COD day^−1^, mainly from fatty acid synthesis as well as biodiesel production. The sample contained mainly propionate (315 mg COD L^−1^), acetate (173 mg COD L^−1^), and glycerol (160 mg COD L^−1^). Samples were stored at −80°C and revived following the protocol as described by Kerckhof et al. (2014) using cryoprotection agents, such as dimethyl sulfoxide, plus trehalose and tryptic soy broth. Enrichment of glycerol degraders was performed in 20 mL Balch tubes using modified homoacetogenic medium (HA medium) as described by Patil et al. (2015a), supplemented with 15 g L^−1^ of glycerol as the only carbon source. The medium was adjusted to pH 6.0 with 0.1M phosphate buffer. The tubes with growth medium were inoculated with a suspension of 10^−3^ dilution of original sludge sample, prepared in miliQ water, and incubated at 28°C for an initial period of 11 days. Following three subsequent transfers (10%, v/v) in freshly prepared medium on a weekly basis, the mixed microbial community was then transferred and further enriched at the same incubation conditions for 20 days in 40 mL penicillin bottles using an autoclaved modified growth medium as described by Dennis et al. (2013). Medium composition: 2 g L^−1^ Na_2_HPO_4_, 1 g L^−1^ KH_2_PO_4_, 1 g L^−1^ NH_4_Cl, 0.0147 g L^−1^ CaCl_2_·2H_2_O, 0.1 g L^−1^ MgSO_4_·7H_2_O and 15 g L^−1^ glycerol. The medium was supplemented with 1 mL L^−1^ of a mixed trace element solution and 1 mL L^−1^ of a vitamin solution (Patil et al., 2015a). During all enrichment experiments, the incubations were sampled on a weekly basis.

### Bioelectrochemical System Setup

**Figure 1:**
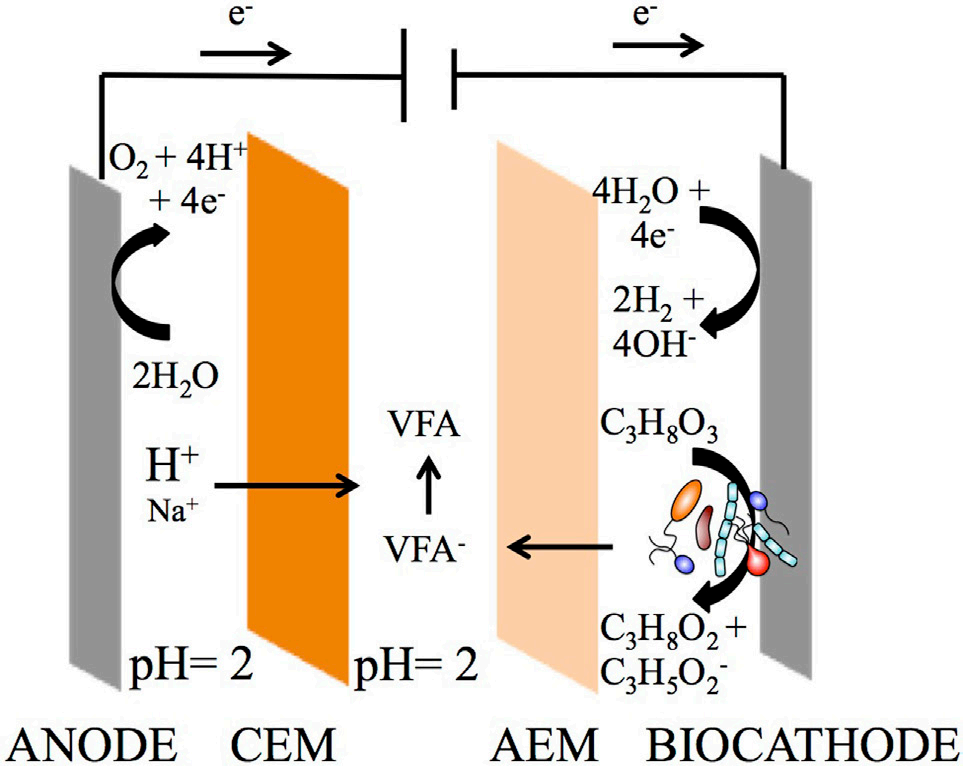
**Three-compartment reactor (3C) concept for simultaneous bioproduction of 1,3-PDO and extraction of carboxylates from glycerol electro-fermentation**. A reduction process occurs at the cathode, converting glycerol into 1,3-PDO and organic acids, whereas an oxidation process occurs at the anode, converting water into oxygen and protons. An anion exchange membrane (AEM) separates the cathode and extraction compartments, allowing the volatile fatty acids (VFA), such as propionate, to cross. A cation exchange membrane (CEM) separates the anode and extraction compartment, allowing the protons to cross, which turn propionate into propionic acid. The extraction (middle) compartment serves the purpose of recovering carboxylates as carboxylic acids.

Two independent BESs were constructed using Perspex frames. A two-compartment system (2C) with a cation exchange membrane (CEM) (Fumatech FKB, Fumasep, Germany) fixed between the cathode and anode compartments was considered as control reactor for 1,3-PDO production as previously described (Zhou et al., 2013). A three-compartment system (3C; Gildemyn et al., 2015; Figure 1), dedicated to 1,3-PDO production and *in situ* organics extraction, was built with an additional AEM (Fumatech FAB, Fumasep, Germany) between the cathode and extraction compartments. Both membranes were pretreated in accordance to the specifications of the company. Acid and base pretreated carbon felt (3 mm thick; 5 × 20 cm exposed to the liquid; Alfa Aesar, Germany) was used as a cathode electrode with a stainless steel frame current collector. An IrOx-coated titanium mesh (5 × 20 cm; Magneto Special Anodes BV, the Netherlands) was used as a stable anode. In the cathode compartment, an Ag/AgCl electrode (3M NaCl, 0.209 V vs. standard hydrogen electrode; RE-1B, Biologic Science Instruments, France) was used as a reference electrode. All electrodes and membranes have a projected surface area of 100 cm^2^. All reactor compartments have a working volume of 350 mL, which includes 150 mL of liquid volume in a closed recirculation buffer vessel of 500 mL. The pH of the cathode was controlled with 2M NaOH and 2M HCl at pH 6.0 (Dulcometer D1Cb/D1Cc, Prominent, Netherlands).

### Bioelectrochemical System Operation

The cathodes were inoculated with a mixed-microbial community enriched with specialized glycerol degraders. The initial concentration of intact cells was set at 4.0 × 10^7^ cells mL^−1^ as determined by flow cytometry according to De Roy et al. (2012). The catholyte was a modified medium as reported by Dennis et al. (2013). The anolyte was 0.1M H_2_SO_4_ (pH 2). The medium in the extraction compartment consisted of four times concentrated inorganic catholyte medium. The catholyte, anolyte, and the extraction compartment liquids were recirculated at a rate of 3.6 L h^−1^ to facilitate mixing. The electrochemical experiments in both BESs were conducted galvanostatically at a fixed current density of 10 A m^−2^ using a VSP potentiostat (Biologic, France). The resulting cell potential was around 2.78 ± 0.07 V throughout the whole operating period. The BESs were operated in a fed-batch mode; 15 g L^−1^ (18.24 g COD L^−1^) of glycerol (min 97% purity, Laborama VWR BDH Prolabo, Netherlands) was added as soon as the glycerol concentration decreased close to 0 g L^−1^. The catholyte, extraction medium, and anolyte were sampled three times per week. Total gas production was determined three times a week by means of a water displacement column assembly. The relative gas composition was determined by analyzing a gas sample from the water displacement column.

### Chemical Analysis

Organic acids and cathode gas composition were analyzed according to Patil et al. (2015a). Alcohols were analyzed according to Gildemyn et al. (2015). Samples for organic acids and alcohol analysis were stored at −20°C and appropriately diluted with milliQ water before analysis. Organic acids (C1-C5) were analyzed using a 930 Compact IC Flex (Metrohm, Switzerland) ion chromatography system with inline bicarbonate removal (Metrohm CO_2_ suppressor). Separation was done on a Metrosep organic acids (250/7.8) column at 35°C behind a Metrosep organic acids (4.6) guard column. The eluent was 1 mM H_2_SO_4_ and the flow rate 0.5 mL min^−1^. An 850 IC conductivity detector was used for detection of eluted components. Detection was enhanced using a chemical suppression module to replace protons with Li-cations (Metrohm suppressor module with 0.5M LiCl). The sample aspiration needle was cleaned with acetone between each analysis. The lower limit of quantification was 1 mg L^−1^ (Gildemyn et al., 2015). Alcohols were analyzed using gas chromatography-flame ionization detector (GC-FID, Varian CP-3800 Gas Chromatograph, USA) during the enrichment phase of the study. The GC-FID has detection limit of 2.0 mg L^−1^ to 1000 mg L^−1^. During the BES experiment phase, alcohols (C1-C4) were analyzed using a 930 Compact IC Flex (Metrohm, Switzerland) ion chromatography system. Separation occurred at 35°C on a Metrosep Carb 2 (250/4.0) column behind a Metrosep Trap 1 100/4.0 guard column. The eluent was 20 mM NaOH at a flow rate of 0.8 mL min^−1^. An IC amperometric detector (cycle: 300 ms 0.05V, 50 ms 0.55 V, 200 ms −0.1 V detection during 200–300 ms of each cycle) was used for detection of eluted components. The sample aspiration needle was cleaned with acetone between each analysis. The lower limit of quantification was 1 mg L^−1^. Standards and controls were regularly used for calibration of the signals (Patil et al., 2015a). Effluent gases from reactors were analyzed on a Compact GC (Global Analyser Solutions, Breda, the Netherlands), equipped with a Molsieve 5A pre-column and Porabond column (CH_4_, O_2_, H_2_, and N_2_) and a Rt-Q-bond pre-column and column (CO_2_, N_2_O, and H_2_S). Concentrations of gases were determined by means of a thermal conductivity detector. The carrier gases were N_2_ for H_2_ channel and He for all other gases. The detection limit for these gases was 100 ppmv.

### Calculations

Coulombic efficiency (CE) and production rate per electrode surface were calculated as previously described by Patil et al. (2015b). Glycerol can be fermented toward hydrogen, therefore CE reported in this work are based on total charge delivered to the cathode through electrical current and glycerol. For example, the overall CE of 1,3-PDO production is calculated according to Eq. 1.

$${\text{CE}}_{1,3\text{PDO}}=\frac{\left(\frac{\left([1,3{\text{PDO}}_{\text{c;t}}]-[1,3{\text{PDO}}_{\text{c;t}-1}])\times V_{c}+([1,3{\text{PDO}}_{\text{m;t}}]-[1,3{\text{PDO}}_{\text{m;t}-1}]\right)\times V_{m}+([1,3{\text{PDO}}_{\text{a;t}}]-[1,3{\text{PDO}}_{\text{a;t}-1}])\times V_{a}}{M_{\text{1,3PDO}}}\right)\times \frac{n_{\text{1,3PDO}}}{A}}{(\left(\frac{J_{\text{app}}}{F}\right)\times 3600\times 24\times (t-t_{-1})+\left(\frac{\left[{\text{Gly}}_{\text{c};\text{t}-1}]-[{\text{Gly}}_{\text{c};\text{t}}\right]}{M_{\text{gly}}}\right)\times \frac{n_{\text{gly}}}{A})}\times \text{100}$$

with CE_1,3-PDO_ as the overall CE of 1,3-PDO production in %, [1,3-PDO_x;y_] as the concentration of 1,3-PDO in g L^−1^ in a certain compartment at a certain time point (time in days), *V*_x_ the volume of the corresponding reactor compartment in L, *M*_z_ the molar mass of the component in g mol^−1^, *n*_z_ the number of electrons contained in component z, *A* the area of the electrode in square metre, *J*_app_ the applied current density in ampere per square metre, F the faraday constant of 96485 C mol^−1^, [Gly_c;y_] as the glycerol concentration in the cathode at a certain time point. This equation takes the occurrence and disappearance of 1,3-PDO in each reactor compartment into account. 1,3-PDO is an uncharged molecule and is able to diffuse along the concentration gradient over the ion exchange membranes. For glycerol, a similar consideration was made, but concentrations were negligible in the middle and anode compartment (~100 mg L^−1^) and, thus, not taken into account in the calculations. The observed low diffusion of glycerol is attributed to fast consumption in the cathode and, thus, the concentration gradient was less strong as compared with the 1,3-PDO concentration gradient. CE was calculated in a similar fashion (i.e., considering an overall production from current and glycerol) for hydrogen production by taking the partial pressure and the gas flow rate into account. A separate efficiency for only current (CE_j_) or glycerol (CE_gly_) as electron donor can also be calculated by considering the other source as 0 in the denominator of Eq. 1.

## Results

### Enrichment of Specialized Glycerol Degraders

**Figure 2:**
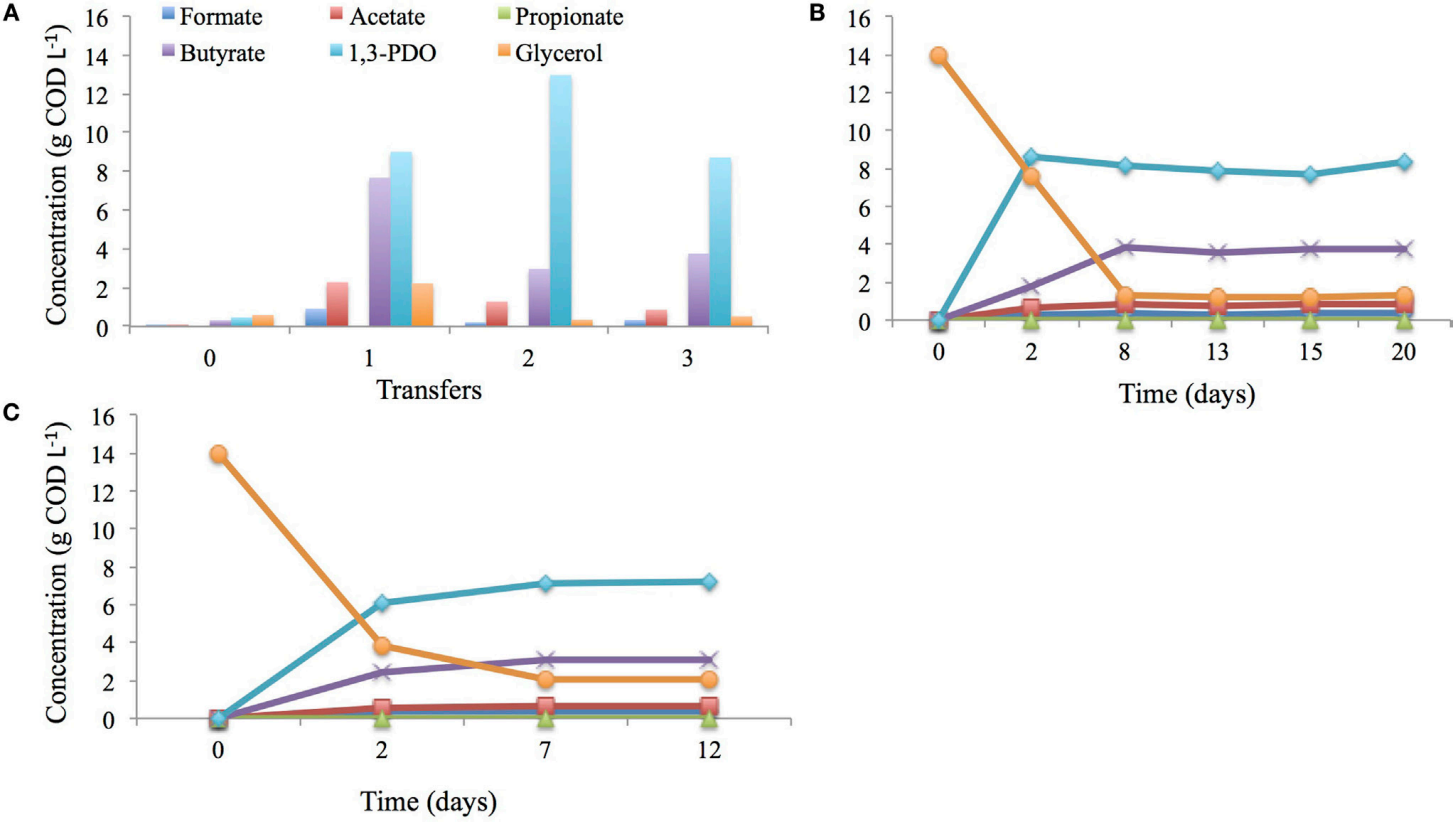
**Enrichment of glycerol degraders at 28°C, pH 6.0 in anaerobic conditions, through three consecutives cycles (highlighted, here, by transfer 1, 2, and 3) of 11 days in 10 mL medium in Balch tubes (A), in 40 mL medium in penicillin bottles for 20 days (B) using HA medium or modified Dennis et al., 2013 medium for 12 days (C)**.

In this study, the microbial inoculum used for bioelectrochemical synthesis of 3-carbon compounds had been through a selection and enrichment cycle. In a first cycle, the mixed-microbial community sample from a wastewater treatment plant of an oleochemical industry was cultured at 28°C and pH 6.0 in minimal medium with glycerol as an only carbon source for a period of 11 days. Mainly 1,3-PDO (0.5 g COD L^−1^) and butyrate (0.3 g COD L^−1^) were produced but glycerol was not entirely consumed (0.6 g COD L^−1^) during this cycle (Transfer 0; Figure 2A). The headspace was dominated by nitrogen (54.2%) and hydrogen (17.8%), in this first cycle, and stayed constant throughout the whole enrichment procedure. Consumed glycerol mainly ended-up in microbial biomass production as dense bacterial growth (visual observation of turbidity) was observed after 11 days of incubation. Following a first transfer (Transfer 1; Figure 2A), microbial growth was still high, but within the same period of time (11 days), 1,3-PDO (9.0 g COD L^−1^) as well as butyrate (7.7 g COD L^−1^) concentrations were considerably higher. However glycerol (2.2 g COD L^−1^) was still detected after 11 days. It was only after a second subsequent transfer (Transfer 2; Figure 2A) into fresh growth medium that glycerol was removed for 98% (0.3 g COD L^−1^), which coincided with a high 1,3-PDO concentration (13.0 g COD L^−1^) and a lower butyrate production (3.0 g COD L^−1^) compared with the previous transfer. A third and last transfer into fresh growth medium did not improve glycerol degradation any further; a decrease in 1,3-PDO (8.6 g COD L^−1^) and a slight increase in butyrate (3.7 g L^−1^) production were detected (Transfer 3; Figure 2A). Additionally, CH_4_ was never detected during the entire enrichment procedure.

Following this pre-enrichment of glycerol degraders, a similar enrichment procedure was performed using a larger volume of medium (40 mL) for 20 days. After 8 days of incubation, glycerol was mainly converted into 1,3-PDO (7.1 g COD L^−1^) and butyrate (3.1 g COD L^−1^). No further conversions occurred as the titers remained constant (Figure 2B).

The enriched mixed-microbial community was then transferred into modified medium from Dennis et al. (2013) to acclimate the community to the BES medium for a period of 12 days. After 7 days of incubation, a large fraction of glycerol (15.8 g COD L^−1^) was consumed and 1,3-PDO production reached up to 8.2 g COD L^−1^ and butyrate to 3.8 g COD L^−1^ (Figure 2C).

### Enhanced Glycerol Recovery into 3-Carbon Compounds in BESs

**Figure 3:**
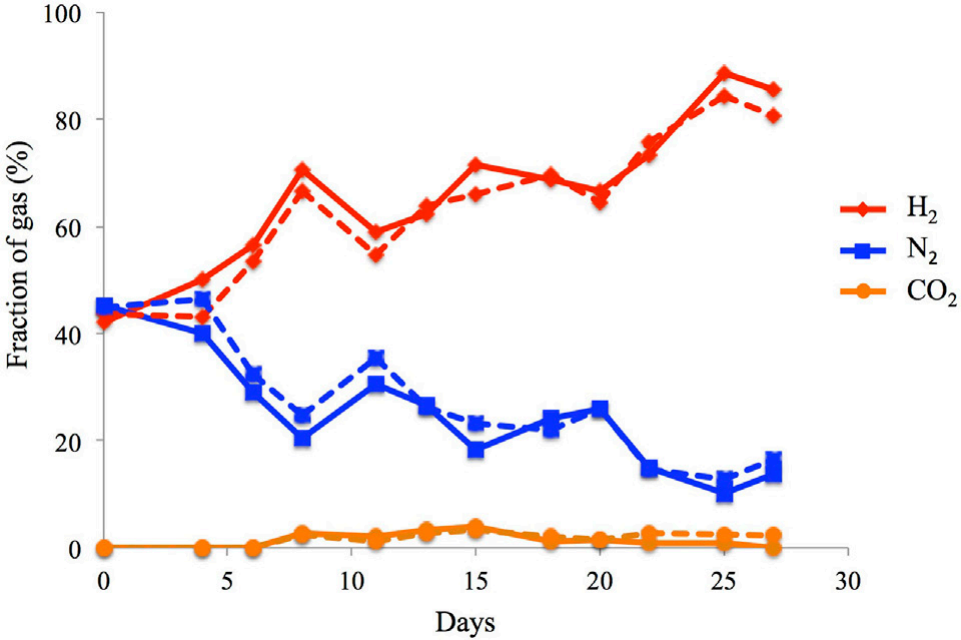
**Gas composition of the cathode as determined from the headspace of a water displacement column in 2C (-) and 3C (- -) bioelectrochemical systems**.

In both reactors, headspace gas composition was very similar to each other (Figure 3). Hydrogen gas dominated the headspace of the reactors in the range of 60–88%, with the remainder being N_2_ (10–38%) and CO_2_ (0.1–4.3%). No methane was detected in the 2C and the 3C reactors, indicating successful inhibition of the methanogenic microbial community during the enrichment phase. The hydrogen production rate was 2.8 ± 0.6 L m^−2^ day^−1^ for 2C and 2.7 ± 0.7 L m^−2^day^−1^ for 3C which corresponds to an overall CE for H_2_ production of 16.7 ± 6.2% for 2C and 14.6 ± 4.6% for 3C (Eq. 1).

**Figure 4:**
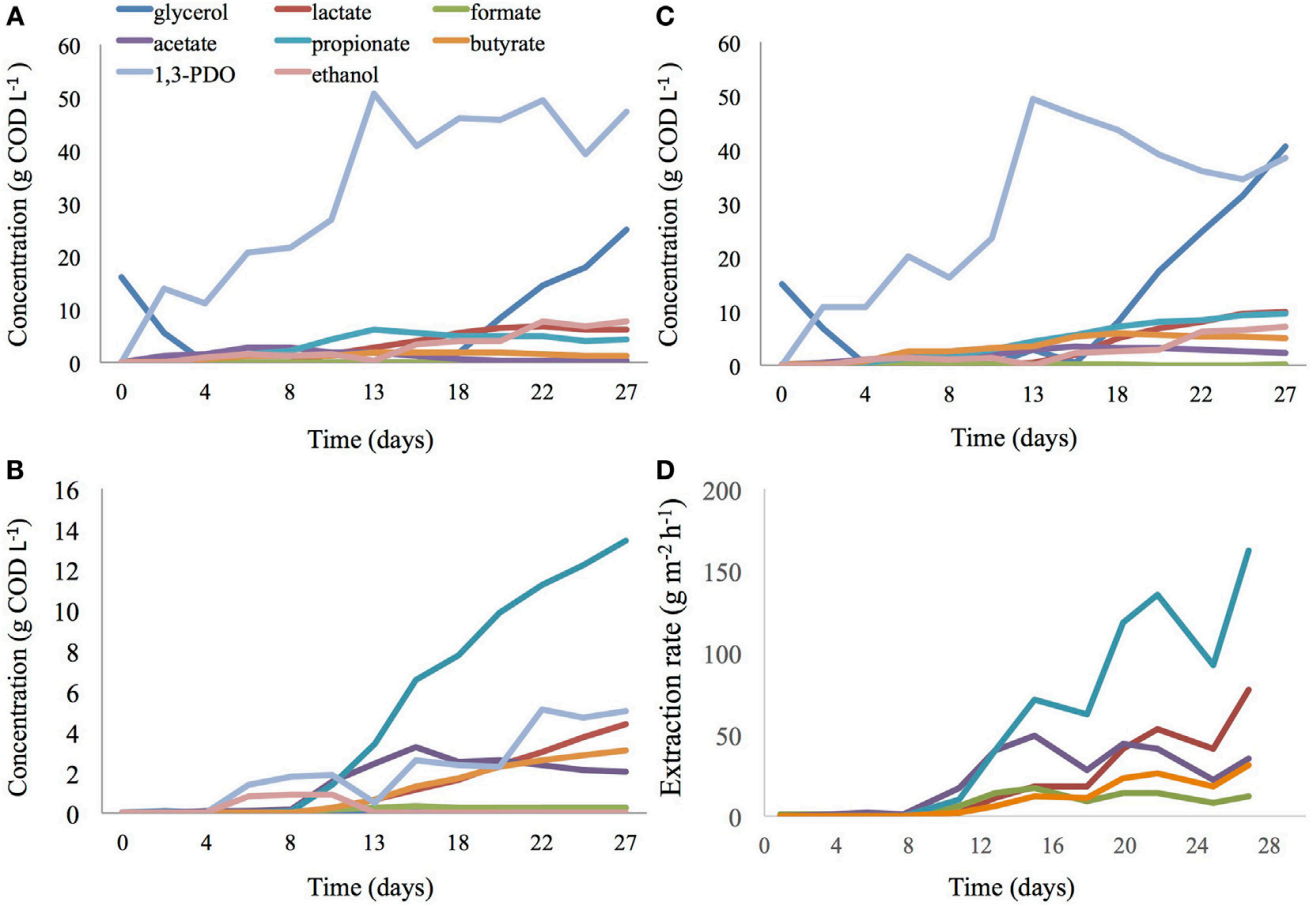
**Glycerol, alcohols, and VFA concentration in the cathodic compartment of a 3C (A) and 2C (C) BESs**. VFA and alcohol concentration in the extraction medium of a 3C BES **(B)** and extraction rate of organic acids in a 3C BES **(D)**.

In both reactors, operated under the same conditions, 1,3-PDO reached similar and high titers, production rates, and CE (Eq. 1). After 13 days, 49.5 g COD L^−1^ of 1,3-PDO (Figure 4C; produced at 33.2% CE and 380.5 g COD m^−2^ day^−1^; per unit projected surface area of electrode) with a yield of 0.72 mol_1,3-PDO_ mol^−1^_glycerol_ was detected in the 2C bioelectrochemical reactor. During the same period, 50.8 g COD L^−1^ 1,3-PDO (Figure 4A; produced at 34.1% CE and 391.0 g COD m^−2^ day^−1^) with a production yield of 0.71 mol_1,3-PDO_ mol^−1^_glycerol_ was detected in the 3C bioelectrochemical reactor. A very low concentration of 1,3-PDO (0.1 g COD L^−1^) and no glycerol were measured during this time in the extraction compartment medium of the 3C reactor, showing that the AEM is impervious to glycerol. From day 15, glycerol started to accumulate in the cathode compartment of both reactors and 1,3-PDO synthesis slowed down.

At day 13, oxidative products from glycerol electro-fermentation were dominated by propionate with 4.3 and 9.6 g COD L^−1^ (6.2 g COD L^−1^ in catholyte and 3.4 g COD L^−1^ in extraction compartment; Figure 4B), respectively, in 2C and 3C reactors. These values represent 31.8 and 47.8%, respectively, of total volatile fatty acids (VFA) present in 2C and 3C reactors. Propionate production in both reactor configurations was rather high compared with what was previously obtained during the enrichment phase of this study. In the 2C reactor, butyrate concentration reached a maximum of 5.8 g COD L^−1^ after 18 days of incubation, in accordance with the maximum titers previously obtained during the enrichment phase. During the same period, in the 3C reactor, the total titer of butyrate was much lower, 3.4 g COD L^−1^ (1.7 g COD L^−1^ in catholyte and 1.7 g COD L^−1^ in extraction compartment; Figure 4B).

From day 11 to day 29 (end of experiment), a considerable increase of organic acids was detected in the extraction compartment with the main component being propionic acid, which increased in concentration from 3.4 to 13.1 g COD L^−1^ in the extraction compartment of the 3C reactor (Figure 4B). After 29 days, propionate had been extracted at a rate of 162 g m^−2^h^−1^ (Figure 4D). During this period, propionate was found to accumulate from 4.3 to 9.5 g COD L^−1^ in the 2C reactor (Figure 4C), whereas its concentration was constant around 4.8 ± 0.4 g COD L^−1^ (*n* = 6) in catholyte of the 3C reactor (Figure 4A). The concentration of total VFA (lactate, formate, acetate, propionate, butyrate) increased from 11.4 to a maximum of 26.6 g COD L^−1^ in catholyte of the 2C reactor, while it stabilized to 12.7 ± 0.8 g COD L^−1^ (*n* = 6) in catholyte of the 3C reactor. At day 29, 9.5 and 17.7 g COD L^−1^ of propionate (from which 13.4 g COD L^−1^ was measured in extraction medium) was measured in the 2C and 3C reactor, respectively. After 29 days, based on VFA considered/detected in the study, the total VFA was extracted with an efficiency of 34.2%. Throughout this period, the concentration of 1,3-PDO remained constant in the 3C reactor, from 51.3 (mainly present in catholyte) to 52.5 g COD L^−1^ (47.4 g COD L^−1^ in catholyte and 5.1 g COD L^−1^ in extraction compartment), representing 58.7 ± 0.1% (*n* = 6) of total COD in the catholyte and middle compartment. In contrast, the 1,3-PDO concentration dropped from 49.5 g COD L^−1^ (77.7% of total COD) to 38.5 g COD L^−1^ (34.2% of total COD) in the 2C reactor.

From day 18 until the end of the experiment, glycerol started to accumulate in the catholyte of both systems. Nevertheless, 25.7 and 46.4 g COD L^−1^ of glycerol was still consumed or degraded in the 2C and 3C reactor, respectively.

## Discussion

In this study, the use of a selected mixed-microbial community, enriched for glycerol consumers, allowed further increase in 1,3-PDO concentrations. Previous work has shown that selective enrichment of a stable performing community was beneficial for instant start-up of a microbial electrosynthesis process and reproducible bioproduction profiles (Patil et al., 2015a).

The enrichment procedure for glycerol degraders shows that, after a week of incubation in HA or modified Dennis et al. (2013) medium, mainly 1,3-PDO and butyrate were produced from glycerol degradation. Removal rates for glycerol were similar between the two media (1.8 vs. 1.9 g COD L^−1^day^−1^). Production rates for 1,3-PDO were slightly higher in HA medium (4.3 vs. 3.1 g COD L^−1^day^−1^), while butyrate production rates were similar between the two media (0.5 vs. 0.4 g COD L^−1^day^−1^). In this case, a decline of microbial diversity due to enrichment does not seem to impact the resilience of the key microbial functional groups responsible for conversion of glycerol into 1,3-PDO and butyrate as previously demonstrated (Wertz et al., 2007).

In both BES, the observed production yields are equal or slightly below the maximum theoretical yields as defined by Zeng (1996); calculated considering fermentation (without hydrogen addition) of glycerol into 1,3-PDO by pure culture of *Clostridium butyricum*. These yields are close to the maximum yield obtained so far with minimal growth medium containing crude glycerol in mixed-culture fermentation (0.76 mol_1,3-PDO_ mol^−1^_glycerol_; Dietz and Zeng, 2014) and much higher than the maximal yield reported so far by electro-fermentation (0.57 mol_1,3-PDO_ mol^−1^_glycerol_; Zhou et al., 2015).

Modification of a BES by addition of an extraction compartment clearly showed a direct impact on glycerol oxidation profiles. The extraction compartment enhances propionate production at the expense of butyrate production. Substantial propionate production from glycerol fermentation has been reported earlier in a BES under several conditions: (1) operation for a period longer than 2 weeks (Dennis et al., 2013), (2) with low current density (1 A m^−2^; Zhou et al., 2015), (3) in case of a BES operated with a variable cathode potential (−0.80 V to −1.10 V), and (4) when electric potential was not sufficient for current production from the start of the experiment (Xafenias et al., 2015).

The observed high VFA concentrations, as reported here in catholyte of the 2C reactor, are well known to inhibit anaerobic digestion process (Hanaki et al., 1981), methanogenesis (Koster and Cramer, 1987), or alcoholic fermentation (Lafon-Lafourcade et al., 1984). Inhibition of methanogenesis and alcoholic fermentation are in this case beneficial processes as this allows to recover as much VFA through extraction as possible.

As a future perspective, we propose to operate a system like the 3C BES, with a residence time of less than 18 days to achieve full removal of glycerol and conversion into 1,3-PDO in the catholyte and to propionic acid in the middle compartment. A residence time below 18 days would lead to low 1,3-PDO diffusion to the middle compartment. The middle extraction compartment can be operated in a batch or as a continuous system. A batch mode operation allows to harvest and recover VFA concentrated from the middle compartment, for example by means of esterification as described in Andersen et al. (2016). Alternatively, the use of the *in situ*-produced 1,3-PDO as the alcohol to drive the esterification can be explored. Another method for recovery of the VFA from the middle compartment can be by means of membrane liquid–liquid extraction (Xu et al., 2015).

## Conclusion

The results obtained during glycerol fermentation in the cathode of BESs confirmed that a high current density (10 A m^−2^) combined with a selectively enriched glycerol-degrading community and *in situ* organics extraction in a modified BES setup allowed to recover 1,3-PDO together with propionic acid from glycerol. An additional benefit of the 3C over the 2C reactor system is the limited organic acid accumulation in the fermentation broth. Separation of the 1,3-PDO from the carboxylates allows for easier downstream processing with the added benefit that the carboxylates can be recovered as their acids from the middle compartment. In this particular case, the acids contained 57.9% of propionic acid.

## Author Contributions

JA and KR conceived the study, participated in its design and coordination, and drafted the manuscript. HR and CA performed the enrichment of glycerol-degrading mixed-microbial communities, constructed and operated the bioelectrochemical systems, and did chemical analysis. HR and JA wrote the manuscript. SP participated in the design of the study and review of the manuscript. All authors read and approved the final manuscript.

## Conflict of Interest Statement

The authors declare that the research was conducted in the absence of any commercial or financial relationships that could be construed as a potential conflict of interest.
